# Ups and Downs of Poised RNA Polymerase II in B-Cells

**DOI:** 10.1371/journal.pcbi.1004821

**Published:** 2016-04-14

**Authors:** Phuong Dao, Damian Wojtowicz, Steevenson Nelson, David Levens, Teresa M. Przytycka

**Affiliations:** 1 National Center for Biotechnology Information, National Library of Medicine, National Institutes of Health, Bethesda, Maryland, United States of America; 2 Laboratory of Molecular Immunogenetics, National Institute of Arthritis and Musculoskeletal and Skin Diseases, National Institutes of Health, Bethesda, Maryland, United States of America; 3 Laboratory of Pathology, Center for Cancer Research, National Cancer Institute, National Institutes of Health, Bethesda, Maryland, United States of America; Princeton University, UNITED STATES

## Abstract

Recent genome-wide analyses have uncovered a high accumulation of RNA polymerase II (Pol II) at the 5′ end of genes. This elevated Pol II presence at promoters, referred to here as Poll II poising, is mainly (but not exclusively) attributed to temporal pausing of transcription during early elongation which, in turn, has been proposed to be a regulatory step for processes that need to be activated “on demand”. Yet, the full genome-wide regulatory role of Pol II poising is yet to be delineated. To elucidate the role of Pol II poising in B cell activation, we compared Pol II profiles in resting and activated B cells. We found that while Pol II poised genes generally overlap functionally among different B cell states and correspond to the functional groups previously identified for other cell types, non-poised genes are B cell state specific. Focusing on the changes in transcription activity upon B cell activation, we found that the majority of such changes were from poised to non-poised state. The genes showing this type of transition were functionally enriched in translation, RNA processing and mRNA metabolic process. Interestingly, we also observed a transition from non-poised to poised state. Within this set of genes we identified several Immediate Early Genes (IEG), which were highly expressed in resting B cell and shifted from non-poised to poised state after B cell activation. Thus Pol II poising does not only mark genes for rapid expression in the future, but it is also associated with genes that are silenced after a burst of their expression. Finally, we performed comparative analysis of the presence of G4 motifs in the context of poised versus non-poised but active genes. Interestingly we observed a differential enrichment of these motifs upstream versus downstream of TSS depending on poising status. The enrichment of G4 sequence motifs upstream of TSS of non-poised active genes suggests a potential role of quadruplexes in expression regulation.

## Author Summary

Accumulation of RNA polymerase II (Pol II) in the promoter proximal region has been proposed to be important for rapid gene activation, but the full regulatory role of Pol II dynamics is yet to be delineated. Defining polymerase poising as a significant enrichment of accumulation of Pol II near the promoter, and comparing Pol II profiles in resting and activated B cells, we found that Pol II poised genes generally overlap functionally among different B cell states and correspond to the functional groups identified for poised genes in other cell types. In contrast, non-poised genes are B cell state specific. Interestingly, the transition from poised to non-poised state was not associated exclusively with B cell activation. We found quite a few genes expressed in resting B cells that transitioned from non-poised to poised state after cell activation including several Immediate Early Genes (IEGs)—genes which are known to be activated transiently and rapidly in response to a wide variety of cellular stimuli. In addition, we observed an enrichment of G4 sequence motifs upstream of TSS of non-poised but active genes, including IEG genes. We propose that formation of quadruplexes upstream of TSS can facilitate Pol II engagement by destabilizing the DNA duplex. Taken together, our analysis of resting and activated B cells allowed us to provide novel insight into the dynamics of Pol II poising.

## Introduction

Transcription of protein-coding genes by RNA polymerase II (Pol II) is a complex, multistep process [1–5]. Potentially each of the transcription steps such as polymerase II recruitment, pre-initiation complex (PIC) assembly, open complex formation, promoter escape, pausing, elongation, and termination provides opportunity for a regulatory action [6]. Until recently it has been assumed that the assembly of the pre-initiation complex and Pol II recruitment are the main regulatory steps [7].

Recent genome-wide chromatin immunoprecipitation (ChIP) studies in human [8–10] and *Drosophila melanogaster* [11,12] cells have shown a high accumulation of Pol II at the 5′ end of genes. Previously, several genes, including HSP70 and Myc [13–16], have been known to harbor promoter proximal paused polymerase whose release regulates Pol II entrance into the productive elongation step; this mode of regulation was assumed to be rare. In those studies, Pol II pausing has been defined formally as an event in which the forward movement of elongation-competent transcription complexes is temporarily stopped owing to template sequence, regulatory factors, or both [1].

Genome-wide studies demonstrated that in addition to Pol II pausing, in some cell types Poll II accumulates at the promoters due to different reasons, some of which could also be regulatory. In particular, Maxwell *et al*. demonstrated that during starvation in *C*.*elegans*, in addition to pausing occurring at active stress-response genes, an inactive ‘‘docked” Pol II accumulates upstream of inactive growth genes [17]. Kouzine et al. showed that promoter melting is another key regulatory step of gene expression in resting B cells [18] and it also leads to accumulation of Pol II in the promoter region. Thus promoter-proximal accumulation of Pol II surveyed, for example, by a ChIP-seq experiment, is clearly not sufficient to determine the precise transcriptional status of Pol II. Bona fide pausing can be observed by experiments such as permanganate sensitivity assays [3,11,12], scRNA-seq [19] [20], GRO-seq [21], or PRO-seq [22].

To make a distinction between bone fide pausing and accumulation of Pol II in Chip-seq experiments, accumulation of Pol II at promoter, independent of its status, is often referred to as polymerase poising [11,23,24]. Following this practice, here we consider Pol II to be poised if its density at the promoter is significantly higher than in the gene body independent of a specific Pol II status. We caution readers that the term “poised” has also been used in literature in other contexts, specifically to denote Pol II phosphorylated on Ser-5 residues (RNAPIIS5P) [25,26] or to denote genes with promoters that comprise a bivalent chromatin domain containing a histone modification associated with transcriptional activation, histone H3 trimethylated at lysine 4 (H3K4me3), along with another associated with transcriptional repression, H3K27me3 (for a review, see [27]).

Pol II poising allows for pre-recruitment of Pol II ahead of gene expression. In particular, it is now broadly accepted that Pol II poising facilitates a rapid response to stimuli [12,28–33]. In agreement, promoter-proximal Pol II poising was shown to be prevalent at genes involved in response to stimuli, immune response, and development [11,12,34–37]. An important group of genes susceptible to fast induction are Immediate Early Genes (IEG)–genes that are activated within minutes from stimuli. Pol II poising has been described for several mammalian IEGs including *JunB*, *cFos*, and *cMyc* [36,38,39]. Analyzing rat neurons, Saha et al. found that several IEGs, such as Arc (also known as activity-regulated gene 3.1), are poised for near-instantaneous transcription by Pol II poising [40]. Similarly, immediate mediators of the inflammatory response were shown to be poised for gene activation through RNA polymerase II stalling [34]. Pol II poising provides an opportunity not only for fast but also for synchronized response to stimuli [41]. However the exact mechanism regulating such synchronization is yet to be elucidated. In particular, not all poised genes are induced in response to stimuli [42–44] thus such synchronization would have to be conditioned on additional factors. In addition, studies of Pol II poising during Drosophila development indicated that *de novo* recruitment of poised Pol II does not occur in a tissue-specific manner, necessitating additional tissue-specific regulation [23].

Given the role Pol II poising is proposed to play in the regulation of gene expression, comparative analysis of cells in different stages provides an important tool for understanding this mode of regulation. Recently, such a comparative GRO-Seq based analysis of mouse embryonic stem cells (ESCs) and mouse embryonic fibroblasts (MEFs) [45] has suggested that the transition of Pol II from the poised to the productive elongation stage of transcription is a major regulated step during early differentiation in mouse cells. In *Drosophila melanogaster*, Gaertner et al. showed that Pol II poised status changes during development consistent with the view that poising prepares genes for future expression [23].

While Pol II poising has been proposed as an important regulator of response to stimuli, no analysis of Pol II poising in one of the most informative settings—mouse resting (RESTB) and activated B cells (ACTB)—has been done. Here, we close this gap by performing a comparative analysis of Pol II poising in these cells. Using a classification of Pol II profiles into three groups, we compared functional enrichment of genes in these classes across cell states. Analysis of resting and activated B cells allowed us to identify and investigate groups of genes whose Pol II profile changes in response to B cell activation and thus to provide novel insight into the role of Pol II poising in gene regulation.

## Results

### Poising Index is, on average, lower in activated B cells than in resting B cells

**Fig 1:**
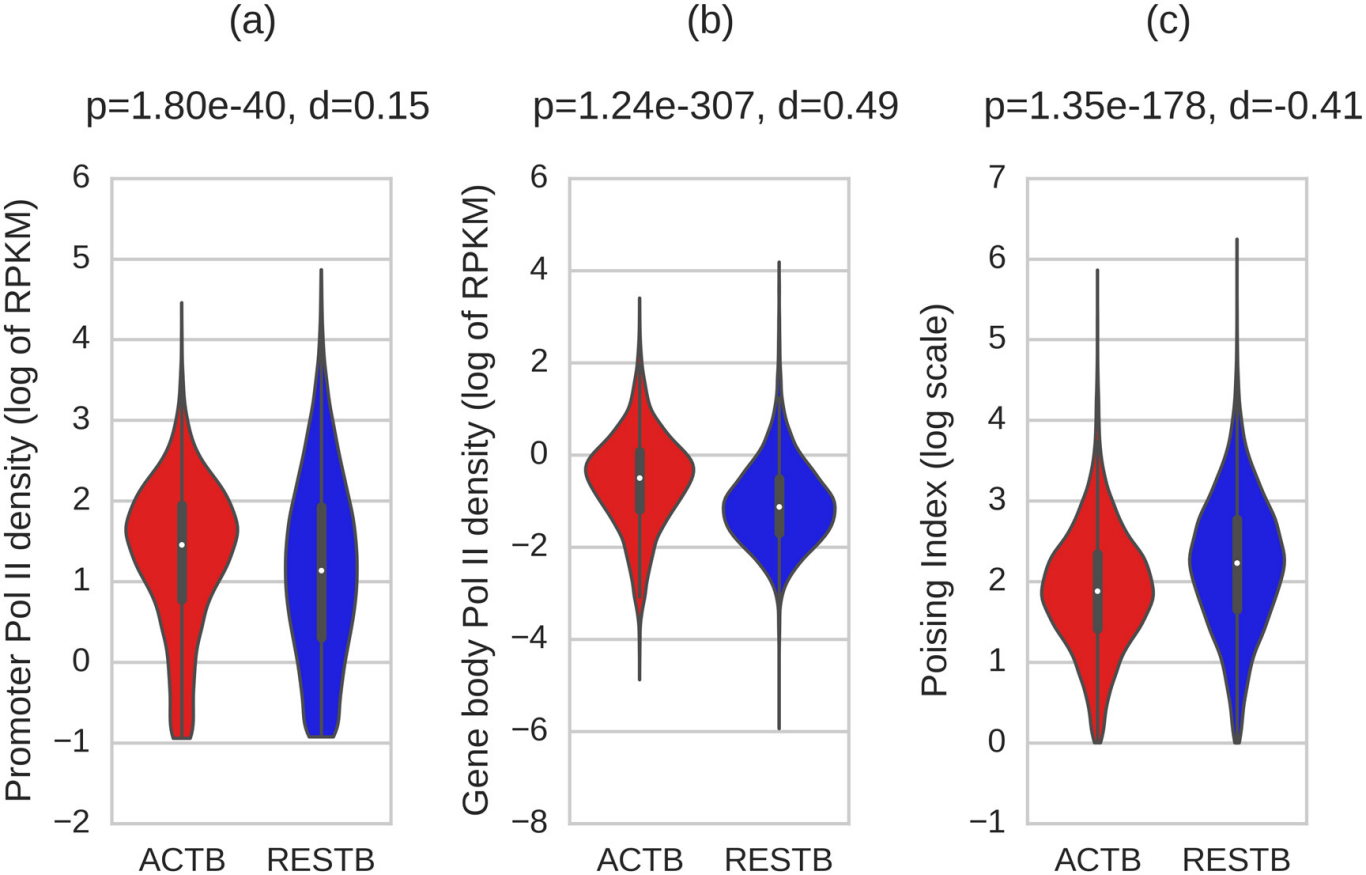
Distributions of Pol II density and its poising index. Violin plots showing (a) the distributions of Pol II promoter density, (b) Pol II gene body density, and (c) poising index values for 9710 genes in ACTB and 9290 genes in RESTB cells with Pol II^+^ promoters. Pol II densities were normalized to reads per kilobase per million reads mapped (RPKM). The y-axes are in a logarithmic scale.

It has been broadly recognized that the distribution of Pol II across gene is not uniform and is often characterized by an overall larger accumulation of Pol II in the promoter region than in the gene body. To examine the Pol II distribution in the analyzed cells, we processed reads from ChIP-seq data of IgG and Pol II as described in Material and Methods section. We considered Pol II to be present at the promoter (Pol II^+^) if the number of Pol II reads at the promoter was significantly higher (p<0.001, Fisher's exact test) than the number of IgG control reads in the same region. Unless otherwise stated, we focused on the Pol II^+^ genes. We use Pol II^−^ to denote genes not in the Pol II^+^ group. Following the well-established practice [11], Poising Index (PI) is defined as the ratio of Pol II density in the promoter to Pol II density in the gene body, as described in Material and Methods section. The distributions of Pol II density in the promoter and gene body regions, and PI values for resting and activated B cells are summarized in Fig 1. The PI distributions differ between two B cell states with the lower PI values in activated B cells (p<1.3e-178, Mann-Whitney U test and Cohen's effect size d = -0.41).

### Pol II poised genes functionally overlap between different cell states while non-poised genes are B cell state specific

We further classified genes with Pol II^+^ promoters based on the relative Pol II density in the promoter and gene body regions. Specifically, if Pol II presence in the promoter is significantly greater than in the gene body, we call these genes Pol II poised genes; otherwise we call them Pol II non-poised genes. The classification was done using Fisher's exact test where we assessed a null hypothesis that the Pol II density in the promoter and gene body is equal (see Material and Methods). In the end, we defined three major classes of genes based on Pol II activity across gene:

- Class I (inactive genes): All Pol II^−^ promoter genes,
- Class NP (non-poised genes): Pol II^+^ promoter and non-poised genes, that is genes with significant Pol II presence in their promoters and with Pol II density in the promoter not significantly greater than Pol II density in the gene body,
- Class P (poised genes): Pol II^+^ promoter and poised genes, that is genes with significant Pol II presence in their promoters and with Pol II density in the promoter significantly greater than Pol II density in the gene body.

**Fig 2:**
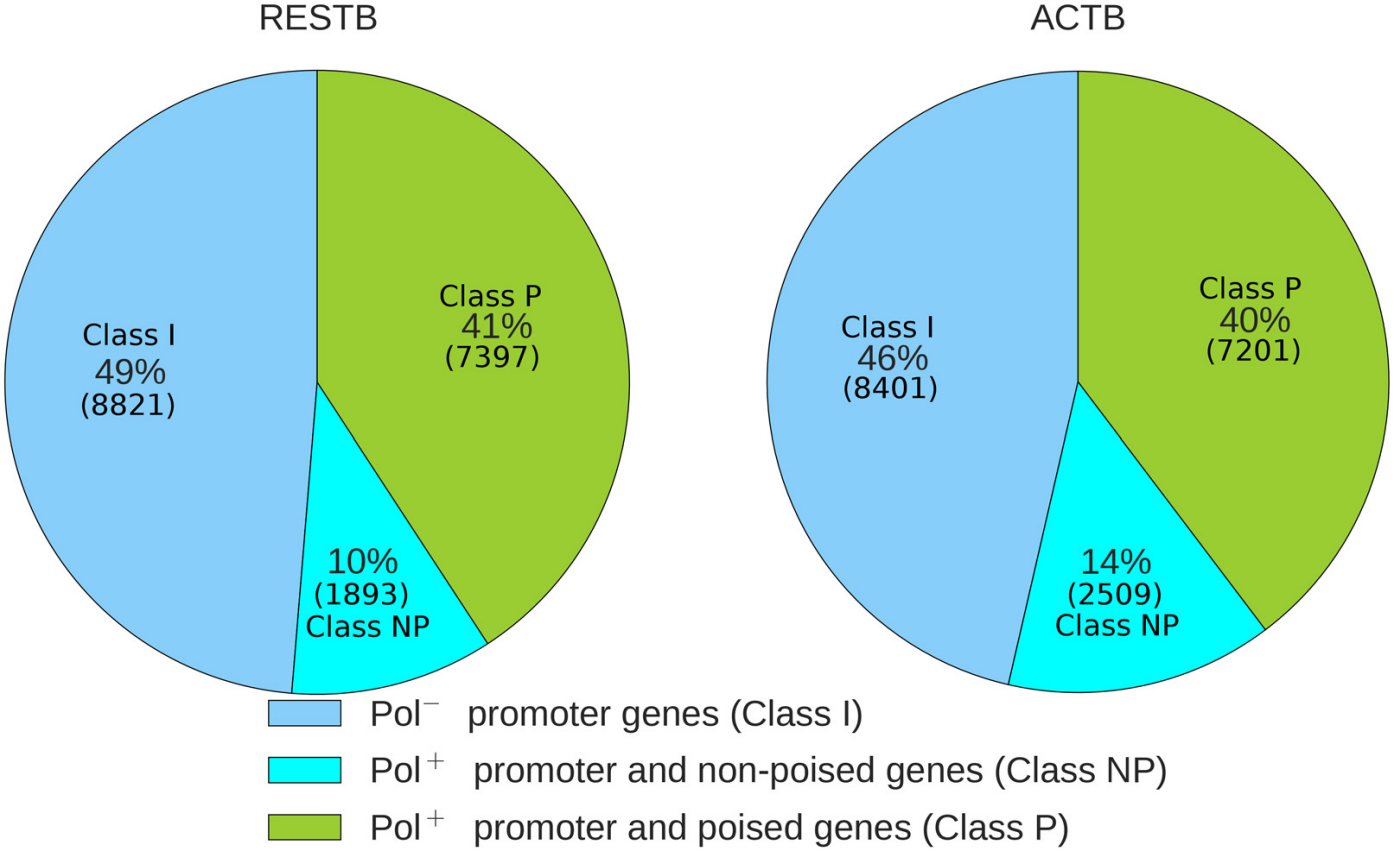
Distributions of gene classes based on Pol II binding profiles. The number of genes in each of Pol II profile classes in RESTB and ACTB cells.

Fig 2 shows the number of genes in each class across different cell states. Both cell states have a similar number of Pol II^+^ promoter genes (51–54% overall): 9710 genes in activated and 9290 genes in resting B cells. However, ACTB have higher number of class NP genes (14% overall and 26% among Pol II^+^ promoter genes).

We next analyzed the functional enrichment of genes in class NP and P in both cell states. Interestingly, considering all genes as a background (Table 1), class P genes are enriched for DNA repair, apoptosis, cell cycle, cellular macromolecule catabolic process, translation, and transcription in both resting and activated B cells. These classes generally correspond to genes that function in response to extracellular or intracellular stimuli [45]. Importantly, the enriched functional categories we identified for B cells largely overlap with categories identified using GRO-seq in mouse embryonic stem cells and mouse embryonic fibroblasts when similarly all genes were taken as the background [45]. This suggests that functional enrichment of poised genes is not only common across different cell stages but also across different cell types. This is consistent with recent results showing that during Drosophila development de novo recruitment of poised Pol II does not occur in a tissue-specific manner [23].

**Table 1:** Biological processes enriched in poised genes with background of all genes.

Enriched processes	ACTB	RESTB
RNA processing	***	***
protein transport	***	***
cellular macromolecule catabolic process	***	***
DNA metabolic process	***	***
DNA repair	***	***
cell cycle	***	***
ncRNA metabolic process	***	***
mRNA metabolic process	***	***
transcription	***	***
translation	**	***
ribonucleoprotein complex biogenesis	**	***
protein folding	**	**
apoptosis	*	**

Enrichment analysis was done with the background of all genes. Stars (*) denote significantly enriched processes while dashes (-) denote non-enriched processes.

*: 1.0 e-05 < p ≤ 0.01,

**: 1.0 e-15 < p ≤ 1.0 e-05, and

***: p < 1.0 e-15. Exact p-values are provided in S1 Table.

In contrast, class NP genes in ACTB (Table 2) are enriched with regulation of lymphocyte activation—a processes that is specific to lymphocyte cells. Thus while Pol II poised genes correspond to the functional groups previously associated with poised genes in other cell types, non-poised genes are B cell state specific. Additionally, class NP genes in ACTB are enriched in translation and transcription. This is consistent with the previous observation that there is an overall 10 fold transcriptional amplification in ACTB relative to RESTB [18]. Gene Ontology (GO) enrichment analysis of class NP genes in RESTB does not show enrichment in any specific GO processes, however, as discussed in detail later, we found that the top genes in this class includes Immediate Early Genes—genes that are relevant for the cell activation process.

**Table 2:** Biological processes enriched in non-poised genes with background of all genes.

Enriched processes	ACTB	RESTB
cell cycle	**	*
regulation of transcription	**	-
regulation of lymphocyte activation	**	-
regulation of cytokine production	*	-
translation	*	-

Enrichment analysis was done with the background of all genes. Stars (*) denote significantly enriched processes while dashes (-) denote non-enriched processes.

*: 1.0 e-05 < p ≤ 0.01,

**: 1.0 e-15 < p ≤ 1.0 e-05, and

***: p < 1.0 e-15. Exact p-values are provided in S1 Table.

To focus more closely on B cell active genes, we additionally performed enrichment analysis using only Pol II^+^ genes as the background. Poised RESTB genes remained enriched in translation and metabolic process (Table 3). Non-poised genes in ACTB remained to be enriched in B cell specific processes and both RESTB and ACTB were enriched in immune response (Table 4). The fact that Pol II poised genes functionally overlapped between different cell states suggests that the tendency of certain groups of genes to be poised is largely context independent, while non-poised and transcriptionally active genes include functional groups that are cell state specific.

**Table 3:** Biological processes enriched in poised with background of Pol II^+^ genes.

Enriched processes	ACTB	RESTB
RNA processing	-	**
mRNA metabolic process	-	**
translation	-	**
protein transport	-	*
cellular macromolecule catabolic process	-	*

Enrichment analysis was done with the background of Pol II^+^ genes. Stars (*) denote significantly enriched processes while dashes (-) denote non-enriched processes.

*: 1.0 e-05 < p ≤ 0.01,

**: 1.0 e-15 < p ≤ 1.0 e-05, and

***: p < 1.0 e-15. Exact p-values are provided in S1 Table.

**Table 4:** Biological processes enriched in non-poised genes with background of Pol II^+^ genes.

Enriched processes	ACTB	RESTB
immune response	*	*
regulation of cytokine production	*	-
regulation of lymphocyte activation	*	-
cell surface receptor linked signal transduction	*	*
G-protein coupled receptor protein signaling pathway	-	*
regulation of lymphocyte activation	**	-
regulation of cytokine production	*	-
translation	*	-

Enrichment analysis was done with the background of Pol II^+^ genes. Stars (*) denote significantly enriched processes while dashes (-) denote non-enriched processes.

*: 1.0 e-05 < p ≤ 0.01,

**: 1.0 e-15 < p ≤ 1.0 e-05, and

***: p < 1.0 e-15. Exact p-values are provided in S1 Table.

### Changes in Pol II profile upon B cell activation

**Fig 3:**
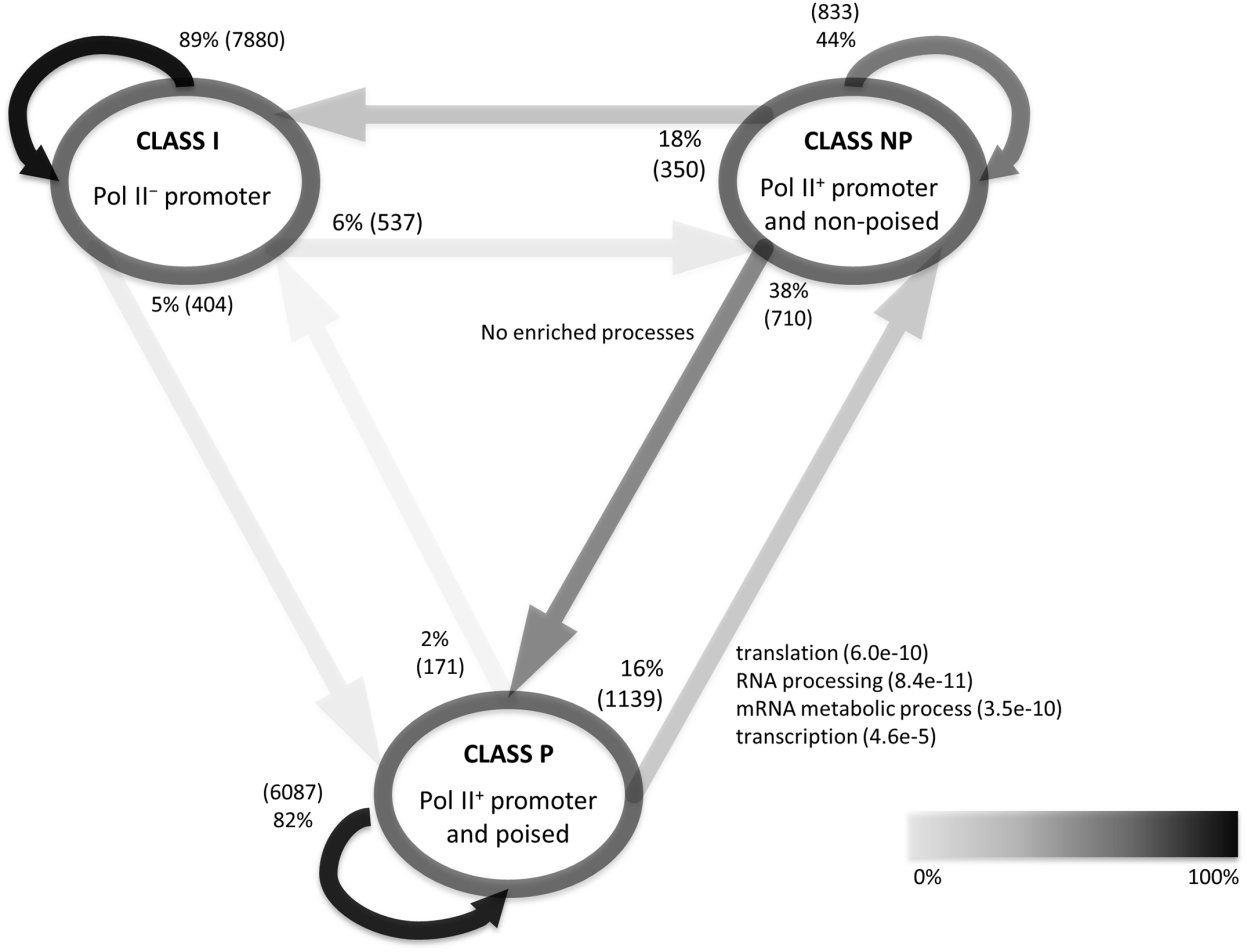
The impact of B cell activation on Pol II profile. Nodes correspond to the Pol II profile classes. Arrows correspond to Pol II profile changes when transitioning from a given profile class in RESTB (arrow start) to a profile class in ACTB (arrow end). The labels along the arrows display the percentage of genes that change the profile class consistently with the arrow. The labels also include GO enrichment terms for the group of genes with the corresponding profile change. GO enrichment analyses were done with the background of all of Pol II^+^ promoter genes in either RESTB or ACTB.

To gain insight into the role of Pol II poising in B cell activation, we asked whether there are any functionally coherent groups of genes that change Pol II profile class after B cell activation. If poising prepares genes for a rapid and simultaneous expression, we might observe an enrichment of certain functional groups within the genes moving from Pol II profile class P to class NP upon B cell activation. GO enrichment analysis of genes that changed Pol II profile from class P to NP (with the background of all Pol II^+^ promoter genes in both RESTB or ACTB) showed that these genes are enriched for cellular processes related to cell cycle and transcription such as translation, RNA processing, and mRNA metabolic process. On the other hand, Pol II^+^ promoter genes non-poised in RESTB (class NP) but poised in ACTB (class P) are not enriched for any specific cellular processes (Fig 3). This observation remains true when Pol II profile change is measured by at least two-fold increase of the PI value rather than by switching profile classes.

**Fig 4:**
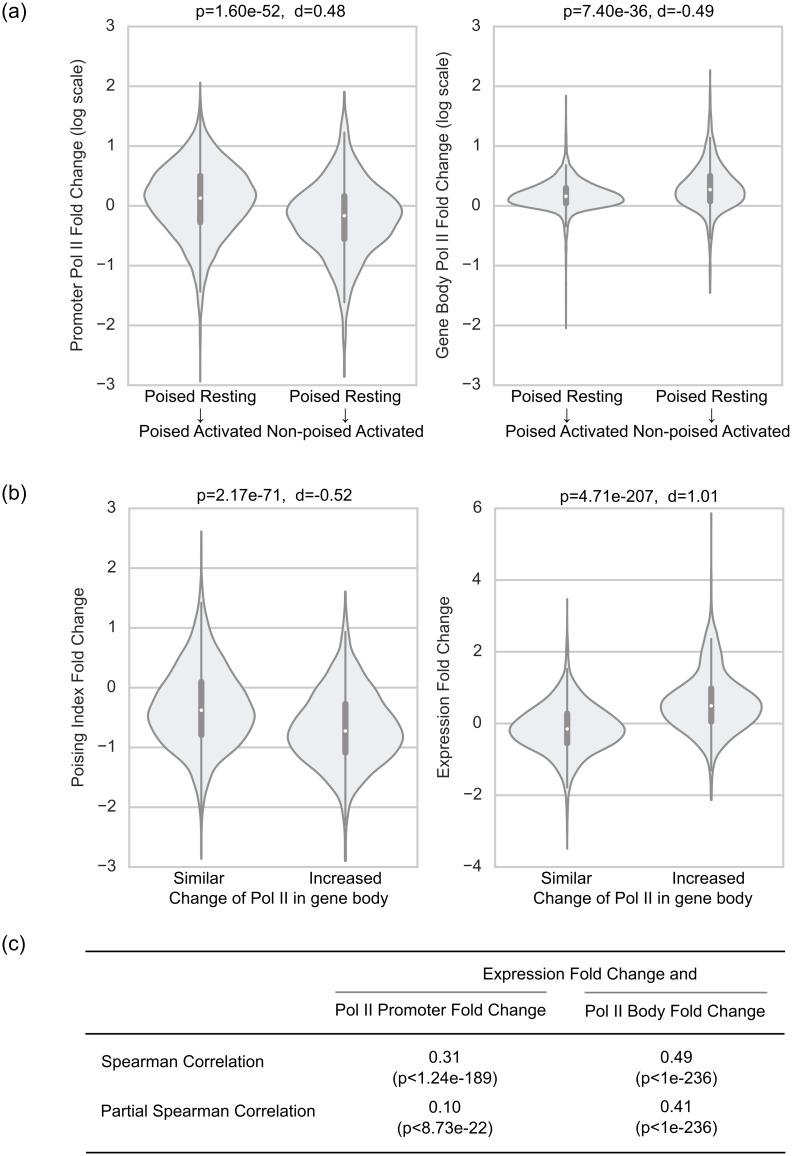
Changes in Pol II profile, gene expression, and poising index upon B cell activation. a) Violin plots comparing distributions of fold changes of Pol II promoter density (left) and Pol II gene body density (right) for two groups genes: genes that continued to be poised after B cell activation (6802 genes) and genes that switch from poised to non-poised class during B cell activation (1138 genes). The y-axes are in a logarithmic scale. The p-values from Mann Whitney U test and Cohen's d effect sizes that reflect the differences between two groups are reported on top. b) Violin plots comparing the distributions of fold changes of poising index and mRNA expression in two groups of genes based on their change in transcriptional activity along gene body upon B cell activation: genes with similar Pol II body density (2/3 < fold change < 1.5; 6069 genes) and genes with large increase of Pol II body density (fold change > 1.5; 1491 genes) between RESTB and ACTB. c) The Spearman and partial Spearman correlations between changes in gene expression and changes in Pol II promoter/gene body density during the transition from RESTB to ACTB for common 8769 Pol II^+^ genes.

We next examined how the change in gene transcriptional activity upon B cell activation relates to the changes in Pol II densities, poising index and expression. The genes that transitioned from poised to non-poised state upon B cell activation where characterized, on average, by increased Pol II density in the gene body and reduced Pol II density at promoters (Fig 4a). In addition, genes with increased density of Pol II in the gene body were characterized by lower poising index and higher mRNA expression (Fig 4b). Given these observations one might expect that there is a correlation between changes in poising index and changes in gene mRNA expression, but there was no such correlation. However, we observed that the genes that transitioned from poised to non-poised state upon B cell activation experienced higher expression increase than the genes that continued to be poised (p<4.2e-7, Mann-Whitney-Wilcoxon test); although the effect size was negligible. Thus we separately examined changes in Pol II density at promoters and gene bodies by asking whether the increase in gene expression upon B cell activation correlates more strongly either with increased Pol II promoter proximal density or with its increased density in gene bodies. We calculated the Spearman and partial Spearman correlations between changes in gene expression and changes in Pol II promoter/body density during B cell activation (Fig 4c). The increase in gene expression upon activation correlated with the increase of Pol II density in both promoter and gene body regions; the correlation was stronger for the increase of Pol II in the gene body even after correcting for the Pol II density change in the promoter. The correlation between the change in gene expression and the change in Pol II promoter density became weak (although statistically significant) after correcting for correlation of mRNA expression change with Pol II change in gene body (see Spearman partial correlation in Fig 4c).

Thus for many genes reduction in poising index value during the transition from RESTB to ACTB can be associated with an increase of Pol II density in the gene body and an increase in gene expression. This is consistent with the enrichment of these genes for cellular processes related to cell cycle and transcription such as translation, RNA processing, and mRNA metabolic process. Indeed activated B cells are much larger and transcriptionally more active than resting B cells [18]. Yet, Fig 4a also indicates that poised to non-poised transition can be a result of reducing of Pol II presence at promoter. Since increased gene expression correlates with increased Pol II promoter density then the increased expression is not the only explanation for reduced poising index. This analysis reveals that while transition from poised to non-poised state is often associated with increased Pol II body density and increased gene expression, change in poising index is not a simple function of expression change.

### Several Immediate Early Genes (IEG) are spontaneously induced and fully elongating in resting cells but poised in activated cells

**Fig 5:**
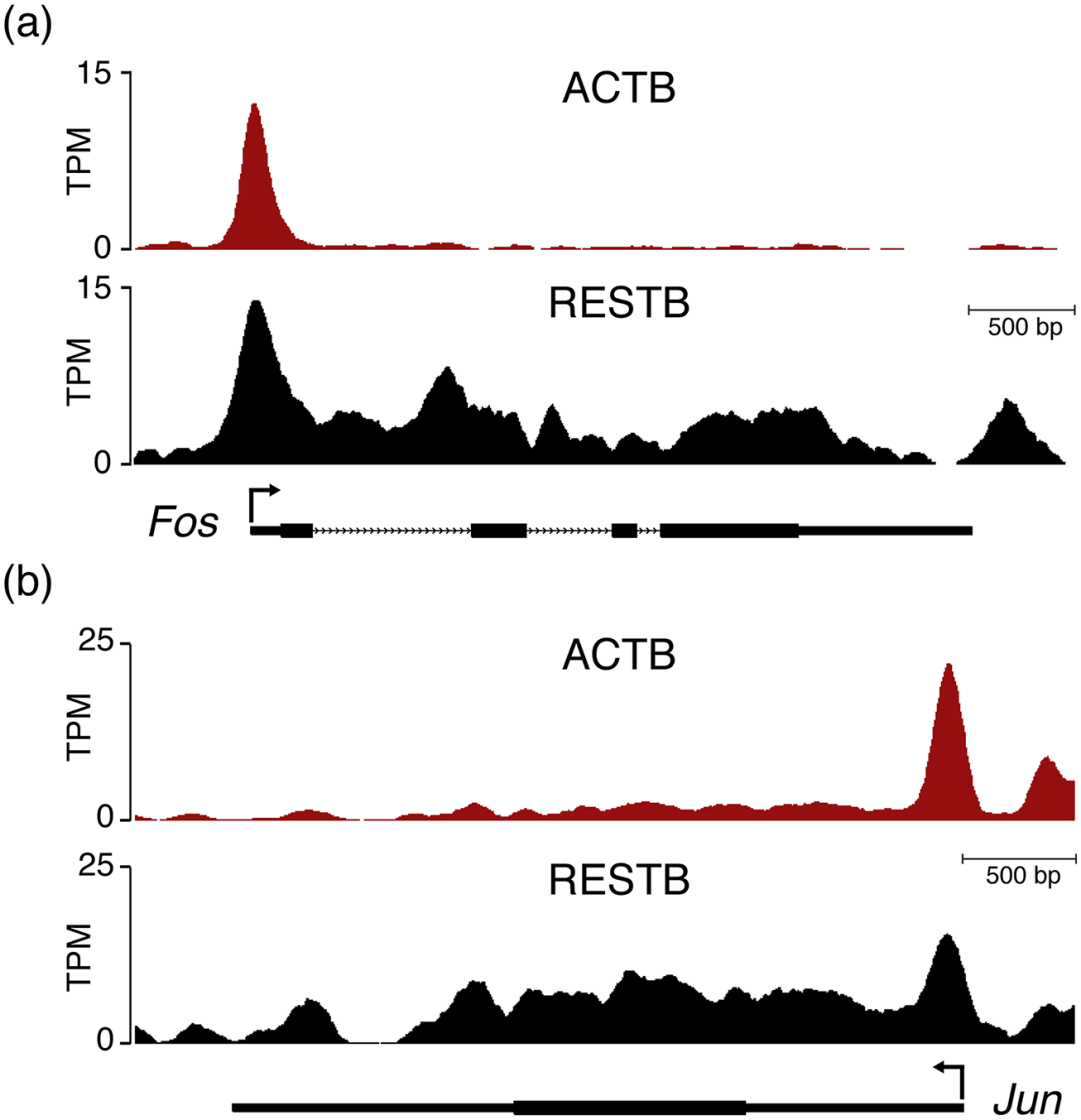
Pol II density profiles of Fos and Jun in resting and activated B cells. Pol II density profiles in the genomic region along (a) Fos and (b) Jun genes in RESTB and ACTB cells. Data were normalized as reads per million reads mapped (TPM).

In resting cells, a majority of genes with Pol II presence at the promoter that are non-poised (class NP) are characterized by low Pol II presence at both the promoter and the gene body. As we showed above, the non-poised genes in resting cells that transition to poised state in activated cells were not enriched in any GO categories. We were interested to see whether such transition can also occurs for genes that are transcribed at significant levels in resting B cells. Sorting them based on Pol II density in the gene body, we found that the top ten of these genes contain known immediate early genes such as *Btg1*, *Fos*, *Jun*, *Egr1*, *Bcl6*, and *Zfp36*. As an illustration, Fig 5 shows the Pol II profile of *Jun* and *Fos* in RESTB and ACTB.

**Fig 6:**
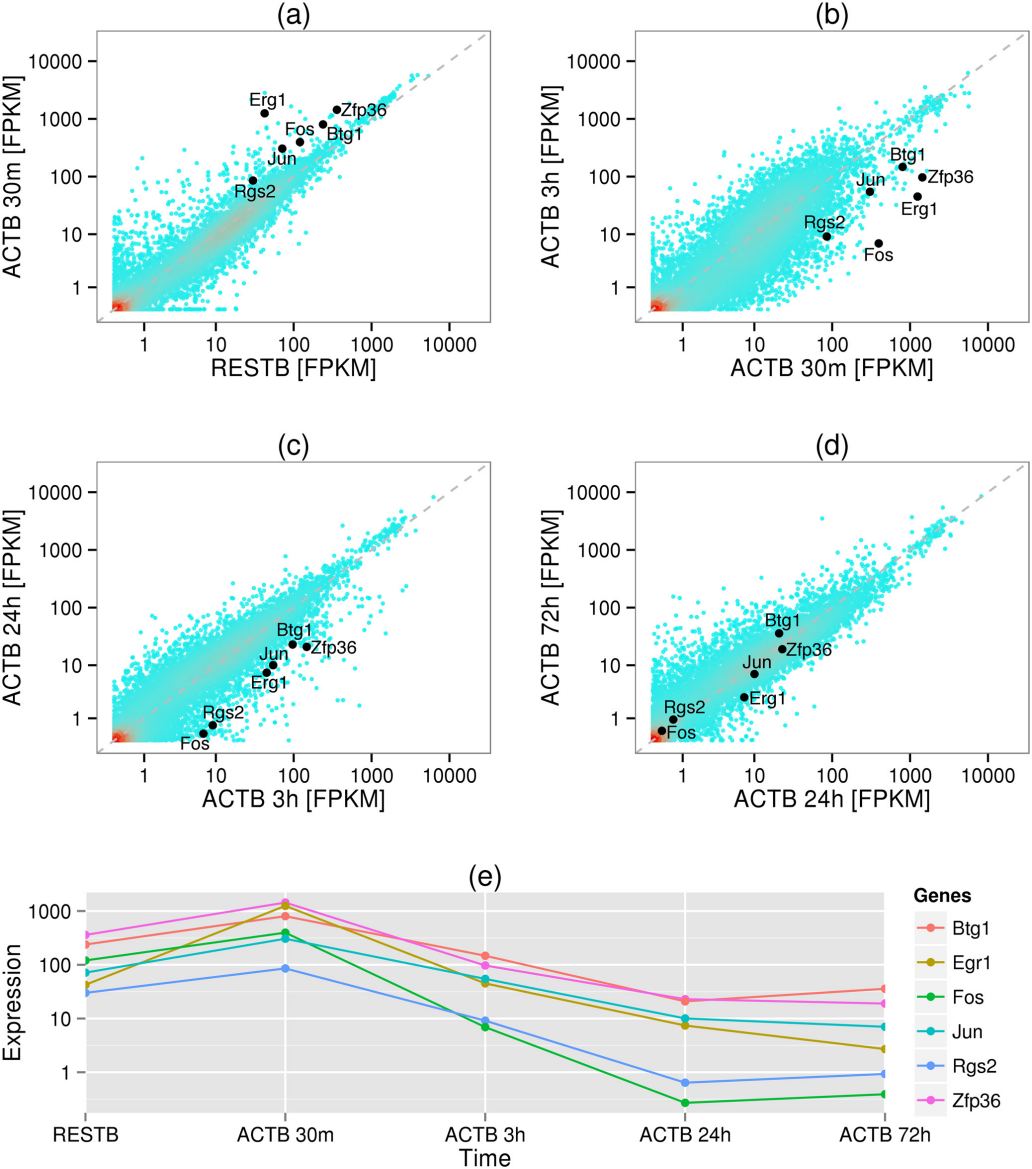
mRNA expression of immediate early genes in resting B cells and calls activated for 30m, 3h, 24h, and 72h. Comparison of mRNA expression levels of all genes (a) in resting B cells and the cells activated for 30 minutes, (b) 30 minutes and 3 hours, (c) 3 hours and 24 hours, and (d) 24 hours and 72 hours. Six immediate early genes are highlighted. (e) Time series of expression levels of six immediate early genes.

We then used measurements of mRNA expression level in resting B cells and in cells activated for 30 minutes, 3, 24, and 72 hours to trace expression dynamics of these genes. We found that their expression increases even further when measured 30 minutes after the B cell activation. Thus despite being highly expressed in resting B cells, these genes have not achieved their full induction level yet. Their expression then gradually drops when measured at 3 and 24 hours after B cell activation and after that remains stable (Fig 6). (We note that our measurements of Pol II in ACTB were done 72h after activation.) Many IEGs have previously been shown to be poised prior to the burst of their expression [39,46,47]. For example, we noted that an immediate early gene, Egr2, is poised and expressed at a very low level in resting B cells and experiences an expression burst 30 minutes after activation. It is possible that the IEGs that we found to be highly expressed in RESTB have been spontaneously activated, which prevented us from observing them in their poised state. Interestingly, our analysis reveals that when the cells are fully activated, the expression of these genes decreases while Pol II accumulates at promoters. Thus after expression burst these IEGs return to poised state.

These results suggest that Pol II poising is not just a regulatory step that prepares genes for sprint. We found that a number of early response genes, including *Jun*, *Fos* and *Egr1*, are highly expressed and non-poised in resting B cells but are poised and have relatively low expression in activated B cells, suggesting that Pol II poising also accompanies attenuation of expression of previously active genes.

### Promoter proximal quadruplex sequence motifs and Pol II poising

**Fig 7:**
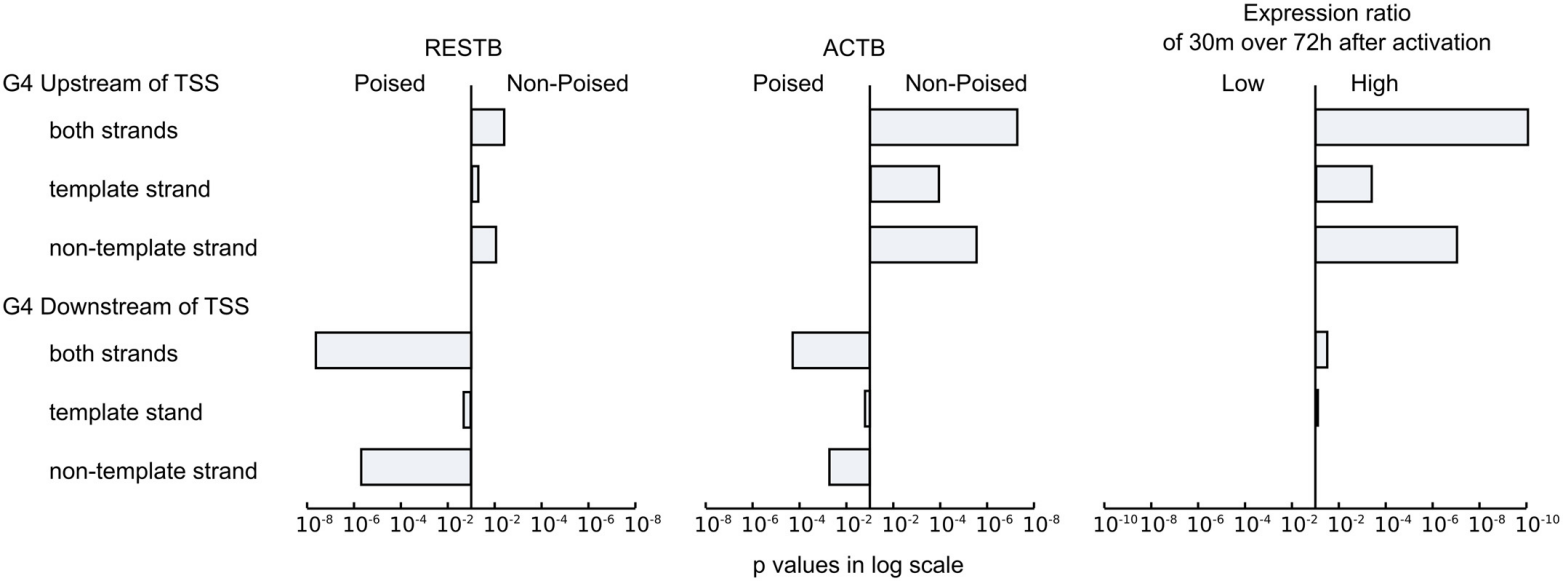
Statistical differences in the number of G4 sequence motifs between poised and non-poised genes, and between high and low ratio of expression at 30m over 72h after cell activation. We plot p-values of the Mann-Whitney-Wilcoxon tests in log scale. The relative position of the bars with respect to the central line indicates enriched category for a given gene group.

Previous studies identified a correlation between the presence of G4 motifs and promoter-proximal poising [48]. Given the observation that for B cell Pol II poising status is cell state dependent, we asked if there is any difference in such promoter proximal sequence motifs between genes with different poising dynamics. G-quadruplexes are non-canonical conformations of DNA molecules. A necessary, but not sufficient, condition for their formation is a particular sequence motif consisting of four runs of G tracks. In this study quadruplex motif occurrences were predicted using QuadParser [49] as described in Material and Methods. Quadruplex motifs are abundant in the mouse genome and are present in the 2kb upstream regions of promoters of up to 50% of the genes. We found that for both RESTB and ACTB cells quadruplexes were enriched downstream of TSS of genes with poised Pol II. Such enrichment was observed before for human cancer NCI-60 cell line and primary T cells [48]. Interestingly we also observed that, as opposed to the poised genes, the non-poised genes in both B cell stages were enriched in these motifs upstream of TSS (Fig 7).

These results suggest that, in the context of Pol II poising, the role of G4 motifs upstream and downstream of TSS is likely to be quite different. Indeed, formation of a quadruplex on template strand can obstruct Pol II movement. In addition, runs of Gs downstream of TSS can be involved in R-loops formation, which was also proposed to facilitate Pol II poising [48], [50,51]. Such R-loops are found to be even more stable if RNA-DNA quadruplexes are involved [52–54]. The enrichment of G4 motifs in downstream region is strongly associated with CpG islands. In contrast the enrichment of G4 motifs in upstream region cannot be explained by the presence of CpG islands (S2 Fig).

Given that the biases with respect to the presence of G4 sequence motifs where similar in both ACTB and RESTB, we asked whether the genes that, similarly to the early response genes, have higher relative expression level shortly after B cell activation as compared to fully activated B cell show any particular bias for the presence of these motifs. We divided the genes into three equal classes: high (HI), medium, and low (LO), depending on the ratio of gene expression in B cells activated for 30 minutes to the expression after 72h of activation. In particular, the genes in the high ratio (HI) group are relatively more actively expressed in the cells shortly after the B cell activation (30m) than in the fully activated cells (72h), as for example the early response genes. Comparing the distribution of G4 motifs in the HI and LO groups we found that the HI group was strongly enriched in G4 motifs upstream of TSS; the enrichment was higher for G4 motifs on the non-template strand than on template strand (Fig 7) suggesting the importance of these motifs for rapid gene expression. Indeed, focusing specifically on early response genes identified above, we found that all of them have G4 motifs upstream of TSS but not downstream.

Overall, our analysis suggests that destabilization of the DNA duplex that accompanies formation of the quadruplexes upstream of TSS can facilitate Pol II engagement and in this way might facilitate rapid progression to elongation for genes that are highly expressed immediately after activation.

## Discussion

Polymerase poising has been defined as a significant enrichment of accumulation of Pol II near the promoter relative to the gene body. This accumulation can be attributed to several mechanisms, including pausing of transcription during early elongation and docking. Such Pol II poising reflects pre-recruitment of Pol II ahead of gene expression. To understand the role of poising for B cell activation, we performed a comparative analysis of Pol II poising in resting and activated B cells. We found that on the genome-wide scale poised genes are consistently enriched for DNA repair, apoptosis, cell cycle, cellular macromolecule catabolic process, translation, and transcription. These functional terms were similar to the previously identified for genes exhibiting Pol II poising in embryonic stem cells and mouse embryonic fibroblasts suggesting that these groups of genes are common across cell types [45]. Also consistently with this view, the genes that change from poised to non-poised as the result of B cell activation have higher relative expression. Focusing on B cell active genes, we additionally performed enrichment analysis using only Pol II + genes as the background. Poised RESTB genes were still enriched in translation and metabolic process. Non-poised genes in ACTB remained to be enriched in lymphocyte activation and both RESTB and ACTB were enriched in immune response—all B cell specific processes. It has been proposed that poising provides a mechanism for synchronized response [1]. Indeed, cellular processes related to cell cycle and transcription such as translation, RNA processing, and mRNA metabolic processing consistently change Pol II profiles from poised to non-poised upon cell activation. Our analysis also supports the contribution of poising to regulation of early response genes. In addition, it provides further evidence to the observation made in the context of Drosophila development that the presence of poised polymerase does not necessarily equate to direct regulation through pause release to productive elongation [55].

Our results indicate that that Pol II poising is not just a regulatory step that prepares genes for sprint. We found that a number of early response genes, including *Jun*, *Fos* and *Egr1*, are highly expressed and non-poised in resting B cells but are poised and have relatively low expression in activated B cells, suggesting that Pol II poising is also associated with previously active genes. A closer analysis of group of genes allowed for identification of interesting relationships between distributions of G4 motifs in poised and non-poised genes. Genes with poised Pol II in both resting and activated B cell where enriched in these motives downstream of TSS while non-poised genes showed upstream enrichment suggesting that the role of G4 motifs in Pol II dynamics might be context dependent. It has been proposed that the runs of Gs downstream of TSS can facilitate Pol II poising by R-loops formation or obstructing Pol II progression [48]. Our results are consistent with such hypothesis. The enrichment in G4 motifs upstream of TSS for non-poised genes has not been observed before. We propose that formation of the quadruplexes upstream of TSS can facilitate Pol II engagement by destabilizing the DNA duplex. Specifically, they could expedite a recruitment of Pol II to the promoter region and accelerate its progression to elongation. Thus, taken together, our analysis of resting and activated B cells allowed us to provide novel insight into the dynamics of Pol II poising.

## Materials and Methods

### Mouse B cell isolation and ex-vivo activation

CD43 negative B cells were isolated from the spleens of 6 to 8 week old C57BL/6J mice (The Jackson Laboratory) by immunomagnetic depletion (Miltenyi Biotech). Activation of the purified B cells was as follows: RPMI-1640 containing FCS, Pen/Strep, glutamine, nonessential amino acids, sodium pyruvate, 2-β-mercapto-ethanol, and HEPES for: 30m, 3h, 24h or 72h in the presence of 25 μg/ml of LPS (E.Coli 0111:B4; Sigma), 5 ng/ml of IL-4 (BioSource) and purified rat anti-mouse CD180 (BD Pharmingen). The cells were incubated at 37 degrees in 5% CO2. At the appropriate aforementioned time points the activated B cells were spun down at 1500 RPM for 5min and resuspended in 1mL of Trizol (Life Technologies) and processed for RNA isolation. Resting B cells were also spun down at 1500 RPM processed in the same manner. Class switching to IgG1 was verified by FACS analysis at 72h.

### Chip-seq and RNA-seq data

We downloaded Pol II binding and IgG control data (ChIP-seq), and mRNA sequencing data (RNA-seq) for resting and activated B cells from the Gene Expression Omnibus under accession number GSE24178 and from the Short Read Archive under accession number SRA072844. For each cell line, all replicates were merged for joint analysis.

### Chip-seq and RNAs-seq data processing

The Chip-seq data with read length from 36 bps to 50 bps were aligned to the mouse reference genome mm9 using Bowtie 2 (version 2.1.0) [56] allowing no more than 2 mismatches and no gaps. We disregarded reads that have multiple best match aligned loci on the reference genome.

We used the spliced read aligner TopHat (version 1.31) [57] to map all RNA-seq reads to the mouse genome (mm9). In order to estimate mRNA expression, we used Cufflinks (version 2.1.1) [58] with the complete set of mouse RefSeq transcripts.

### Gene Ontology (GO) enrichment analysis

We used NCBI’s DAVID software [59] to perform GO analysis. To summarize and remove the similar GO terms, we used REVIGO [60] with the similarity parameter set to medium.

### Gene annotations

We downloaded RefSeq gene coordinates for mouse mm9 assembly from UCSC Genome Browser. Only protein coding genes were considered in our analysis i.e. we kept only transcripts with tag NM. We also filtered out genes that were shorter than 2kb. For each cluster of overlapping genes, we only kept the gene producing the longest transcript and disregard the rest. After these filtering steps, we obtained 18,211 protein coding transcripts.

### Poising index of RNA polymerase II

For each gene, we defined its promoter region as a genome segment from 100 bp upstream to 500 bp downstream of its transcription start site. The body region of a gene starts from 1kb downstream of its transcription start site to its transcription termination site. For each gene, the promoter density of Pol II was calculated as the number of reads aligned to its promoter region normalized to reads per kilobase per million reads mapped (RPKM). Similarly, the gene body density of Pol II was computed using RPKM values. The poising index was computed as the ratio between promoter density and gene body density [59].

### Identification of genes with active promoters and Pol II poised genes

In our analysis, we were interested in genes with significant Pol II binding in their promoters. These Pol II^+^ promoter genes have a significantly higher number of Pol II reads than the average number of IgG reads in the promoter region. Given that the total number of reads is large and the lengths of the promoter/body regions are small compared to the length of the genome, we assume that the number of reads in a segment in the IgG control experiment follows a Poisson distribution. For each gene, we evaluated the null hypothesis that its Pol II promoter density is equal to average IgG promoter density using a one sample Poisson test, with p-values from multiple tests being adjusted using Benjamini and Hochberg's procedure [61]. If p-value< 0.01, the gene has Pol II^+^ promoter; otherwise it has Pol II^-^ promoter.

Among Pol II^+^ promoter genes, we further checked whether they have poised Pol II in their promoters by assessing whether the Pol II promoter density is significantly greater than gene body density. First, we estimated the average IgG promoter and gene body density from the control experiments. Similarly, we calculated the average Pol II promoter and gene body density. For each gene, we formed 2x2 contingency with the rows corresponding to the Pol II and IgG experiments and the columns corresponding to promoter and gene body density. Using Fisher's exact test as in [45], we then assessed the null hypothesis that the Pol II in the promoter and gene body is equal. The p-values were corrected using Benjamini and Hochberg's procedure [61] with the significance threshold of 0.001.

### Comparing enrichment of non-B DNA forming sequences

Non-B DNA forming sequences were identified within 3.0 kb region upstream of TSS or within 500bp region downstream of TSS. Regions with propensity to form quadruplex were predicted using QuadParser program [49] with at least 3 G bases in each of four runs of G repeat and gap size between 1 and 7 nucleotides; both strands were searched. Overlapping regions were merged into a single region.

For each gene, we computed the numbers of quadruplex forming regions; and for each gene group as in Fig 7 and S2 Fig, we calculated median and median absolute deviation of these numbers. Statistical differences between groups were computed using Mann-Whitney-Wilcoxon test.

## Supporting Information

S1 Fig Distributions of Pol II density and its poising index for replicates of ACTB and RESTB. Violin plots showing (a) the distributions of Pol II promoter density, (b) Pol II gene body density, and (c) poising index values in three ACTB and two RESTB replicates; there are 9710 genes in ACTB and 9290 genes in RESTB with Pol II^+^ promoters. Pol II densities were normalized to reads per kilobase per million reads mapped (RPKM). The y-axes are in logarithmic scale.
(TIFF)

S2 Fig Statistical differences in the number of G4 sequence motifs between poised and non-poised genes, and between high and low ratio of expression at 30m over 72h after cell activation. We removed all G4 sequences motifs overlapped with any CpG island. We plot p-values of the Mann-Whitney-Wilcoxon tests in log scale. The relative position of the bars with respect to the central line indicates enriched category for a given gene group.
(TIFF)

S1 Table Biological processes enriched in poised and non-poised genes in activated and resting B cells. Enrichment analysis was done with two different backgrounds: 1) Pol II^+^ genes and 2) all genes. The exact p-values are provided. In brackets we show the number of genes in each group.
(XLSX)
