# Supplementary figures and images for: Ups and Downs of Poised RNA Polymerase II in B-Cells
Source: PLoS Comput Biol. 2016 Apr 14;12(4):e1004821. doi: 10.1371/journal.pcbi.1004821 (PMC4831825; doi:10.1371/journal.pcbi.1004821)

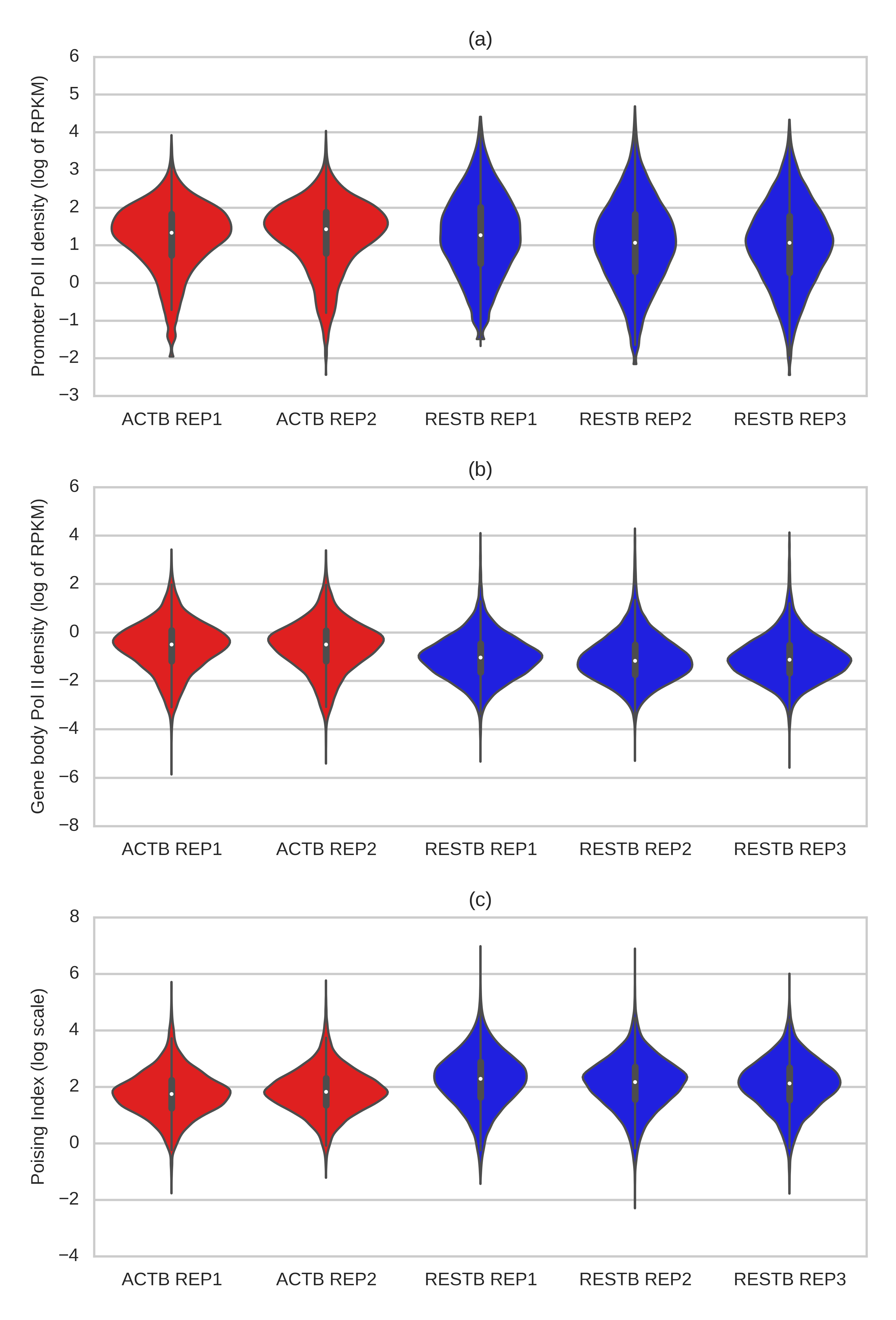

Supplement: S1 Fig — Violin plots showing (a) the distributions of Pol II promoter density, (b) Pol II gene body density, and (c) poising index values in three ACTB and two RESTB replicates; there are 9710 genes in ACTB and 9290 genes in RESTB with Pol II+ promoters. Pol II densities were normalized to reads per kilobase per million reads mapped (RPKM). The y-axes are in logarithmic scale. (TIFF) [file pcbi.1004821.s001.tiff]

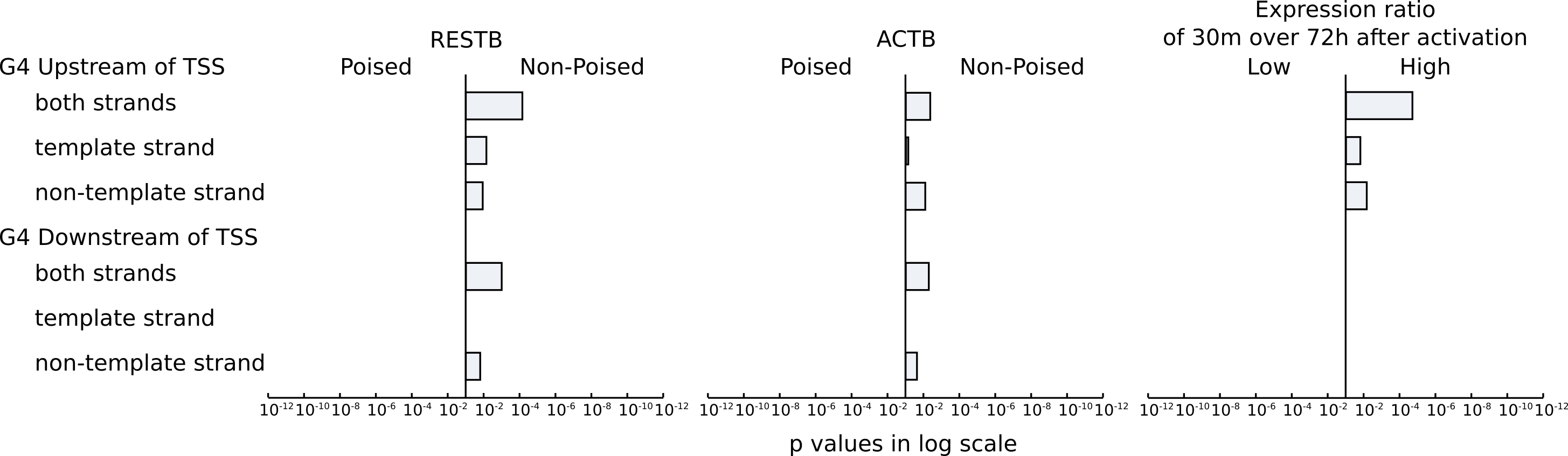

Supplement: S2 Fig — We removed all G4 sequences motifs overlapped with any CpG island. We plot p-values of the Mann-Whitney-Wilcoxon tests in log scale. The relative position of the bars with respect to the central line indicates enriched category for a given gene group. (TIFF) [file pcbi.1004821.s002.tiff]
